# Puerarin—A Promising Flavonoid: Biosynthesis, Extraction Methods, Analytical Techniques, and Biological Effects

**DOI:** 10.3390/ijms25105222

**Published:** 2024-05-10

**Authors:** Sergio Liga, Cristina Paul

**Affiliations:** Biocatalysis Group, Department of Applied Chemistry and Engineering of Organic and Natural Compounds, Faculty of Industrial Chemistry and Environmental Engineering, Politehnica University Timisoara, Vasile Pârvan No. 6, 300223 Timisoara, Romania; sergio.liga96@gmail.com

**Keywords:** puerarin, flavonoids, extraction methods, analytical techniques, biological effects

## Abstract

Flavonoids, a variety of plant secondary metabolites, are known for their diverse biological activities. Isoflavones are a subgroup of flavonoids that have gained attention for their potential health benefits. Puerarin is one of the bioactive isoflavones found in the Kudzu root and *Pueraria* genus, which is widely used in alternative Chinese medicine, and has been found to be effective in treating chronic conditions like cardiovascular diseases, liver diseases, gastric diseases, respiratory diseases, diabetes, Alzheimer’s disease, and cancer. Puerarin has been extensively researched and used in both scientific and clinical studies over the past few years. The purpose of this review is to provide an up-to-date exploration of puerarin biosynthesis, the most common extraction methods, analytical techniques, and biological effects, which have the potential to provide a new perspective for medical and pharmaceutical research and development.

## 1. Introduction

For many centuries, various cultures have used medicinal plants to treat ailments and enhance overall health. These plants contain bioactive compounds that have therapeutic properties, making them a valuable source of medicine [1,2,3,4,5,6]. From ancient civilizations to modern times, the knowledge of medicinal plants has been passed down through generations, contributing to the development of traditional medicine systems such as Ayurveda, traditional Chinese medicine, and Native American healing practices [7].

The popularity of medicinal plants has increased recently due to their natural origin and their potential to have fewer side effects than compounds from synthetic origin [8,9,10]. As a result, extensive research is being conducted to identify and understand the bioactive compounds in these plants, leading to the development of new pharmaceuticals and nutraceuticals [9,11,12].

Today, a significant number of pharmaceutical drugs come from plants or are inspired by the bioactive compounds found in medicinal plants. Flavonoids and phenolic compounds are just some of the many plant secondary metabolites that can be included in bioactive compounds [13,14]. They have the ability to treat numerous health conditions, such as respiratory disorders, digestive problems, skin problems, and chronic diseases like diabetes and cardiovascular conditions [15,16,17].

Flavonoids have received significant attention in nutrition, the medical field, and pharmaceutical research due to their health-promoting effects. Flavonoids are a class of polyphenolic compounds found in various fruits, vegetables, different microorganisms, and medicinal plants [17,18,19]. The diversity of flavonoids in nature and their promising bioactivities make them promising candidates for developing novel therapeutic agents. Their biological activities, which include antioxidant, anti-inflammatory, antiviral, anti-cancer, and antimicrobial properties, have earned them their reputation [17]. Furthermore, flavonoids and other bioactive compounds present in medicinal plants can be used to enhance their therapeutic potential through synergistic effects [20]. Flavanones, flavones, isoflavones, flavonols, flavanols, and anthocyanins are among the subclasses that they further classify into, with each having specific biological effects [17,21,22]. The focus of research studies is now on discovering, extracting, and isolating new plant molecules that have various biological effects, including various high-potential isoflavones.

**Figure 1:**
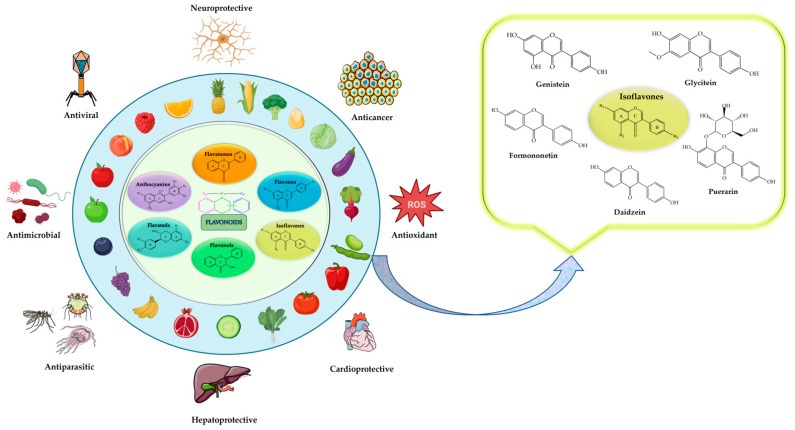
A schematic illustration of the basic structure, natural sources, and biological effects of flavonoids, including the most common isoflavones.

Plant-derived flavonoids known as phytoestrogens are isoflavones, which have structural similarities to the hormone estrogen [23,24,25]. Their natural abundance is present in soybeans and other legumes, and they have been investigated for their potential health benefits [25,26,27]. Due to their potential to have both estrogenic and antiestrogen effects in the body, the phytochemistry of isoflavones is gaining much attention. Their dual biological effects are due to their ability to bind to estrogen receptors, mimicking estrogen’s actions in certain tissues while blocking its effects in others [24,27]. The most common isoflavones are genistein, daidzein, glycitein, and formononetin (Figure 1).

Recently, one of the isoflavones that were discovered has revealed significant therapeutic potential for both the pharmaceutical industry and the entire scientific medical world.

**Figure 2:**
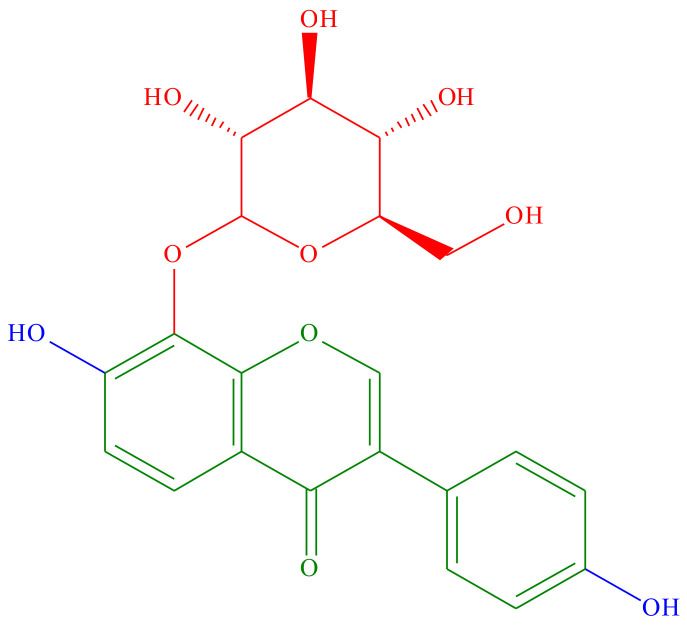
Chemical structure of puerarin.

Puerarin, also known as daidzein-8-C-glucoside, is found in the roots of the kudzu plant and the genus *Pueraria* [28,29,30]. The kudzu plant has compounds like flavonoids, saponins, xanthones, lignans, sterols, and other compounds. The genus *Pueraria* is identified by puerarin, an isoflavone that is used as its chemotaxonomic marker. Isoflavone glycosides, particularly puerarin, are responsible for many of the genus *Pueraria* bioactivities [30]. At positions 7 and 4’, there are hydroxy group substitutions, and at position 8, it is accompanied by a beta-D-glucopyranosyl residue through a C-glycosidin linkage (Figure 2). To develop new applications and improve its bioavailability, it is essential to comprehend the biosynthesis pathway of puerarin.

This review aims to explore the biosynthesis pathway, extraction methods, analytical techniques, and provide a comprehensive summary of the biological effects of puerarin.

## 2. Biosynthesis of Puerarin

Isoflavones are derivatives of flavonoids, which are derivatives of 2-phenyl-benzo-γ-pyrone (2-phenyl-3,4-dihydro-2H-1-benzopyran-4-one). They are included in the large family of natural polyphenolic compounds with structure type C_3_-C_6_-C_3_ [17,31].

Several enzymes and key reactions are involved in the puerarin biosynthesis pathway, which starts with the shikimate pathway. Chorismic acid is formed as the end product of the shikimate pathway after aldol condensation reactions between phosphoenolpyruvic acid and D-erythrose 4-phosphate [17,31]. The enzymes prephenate-aminotransferase (PhAT) and arrogate-dehydratase (ADT) are responsible for converting this into the amino acid phenylalanine. After the formation of the amino acid phenylalanine, biosynthesis occurs through the phenylpropanoid pathway. The deamination of phenylalanine to form trans-cinnamic acid occurs in the presence of phenylalanine-ammonia liase (PhaAL) [17,32]. The 4-coumaric acid is converted from trans-cinnamic acid by cinnamate-4-hydroxylase (C4L). Providing the compound 4-coumaroyl-CoA will be achieved by using 4-coumarate-CoA-ligase (C4CoAL) [17,31,32]. Afterwards, 4-coumaroyl-CoA is converted to isoliquiritigenin by chalcone synthase and chalcone reductase [33,34,35,36,37,38]. Chalcone isomerase (CHI) then catalyzes the formation of liquiritigenin from isoliquiritigenin, which is further catalyzed by 2-hydroxyisoflavanone (IFS) to produce 2,7,4′-trihydroxyisoflavonone [36,37,39].

**Figure 3:**
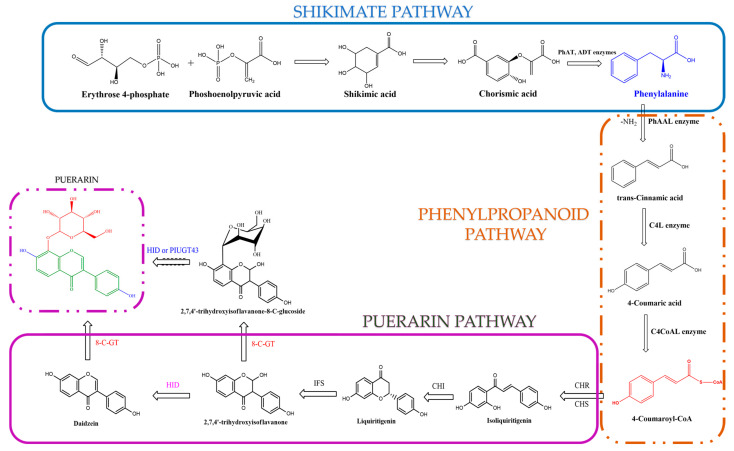
Overview of the main steps of the puerarin biosynthesis pathway.

Two different pathways allow for the production of puerarin through the catalysis of the chalcone isoflavone, by (i) 2-hydroxyisoflavanone dehydratase (HID) to form daidzein, and by (ii) 8-C-glucosyltransferase (8-C-GT) to form trihydroxyisoflavonone-8-C-glucoside (Figure 3) [35,36]. The transformation of it into puerarin is accomplished by PlUGT43 through 8-C-glucosylation. The 8-C-glycosylation reaction during the biosynthesis of puerarin is still being debated [37,40].

## 3. Extraction Methods and Analytical Techniques

Puerarin can be found in several natural sources, such as Kudzu root (*Pueraria lobata*), a traditional medicinal legume taxon native to Southeast Asia, which has a wide range of species and can be found in China [41,42,43]. Additionally, puerarin can also be found in other plants belonging to the genus *Pueraria* [41,42,44,45]. Puerarin can be obtained for various applications using these natural sources in a sustainable and environmentally friendly way.

There are several methods for extracting puerarin from Kudzu root, including the following: (i) solvent extraction; (ii) ultrasound extraction; (iii) enzyme-assisted extraction; (iv) microwave-assisted extraction [46]. Choosing the right extraction method for isoflavones requires considering factors like efficiency, cost, and environmental impact, as each method has its own advantages and disadvantages [17,47,48].

Despite its simplicity and cost-effectiveness, solid-liquid extraction is a traditional method that may not achieve the highest purity of puerarin. Supercritical fluid extraction is a method that offers high purity and efficiency, but it can be expensive and complex to set up [49]. Microwave-assisted extraction can enhance efficiency by accelerating the ex-traction process and reducing extraction time. Using microwave energy can lead to an increase in operational costs [50]. Besides these methods, there have been attempts to extract puerarin using other extraction methods, such as ultrasound-assisted extraction and enzymatic extraction [46].

He Zhu et al. conducted a study to evaluate how differences in ultrasonic power, microwave power, and time affect the rate of flavonoid extraction from Kudzu root samples. Their research revealed that flavonoid extract yield was increased by increasing ultrasonic and microwave power. Microwave power, followed by ultrasonic time and power, were found to be the most effective combination factors for influencing the flavonoid extraction rate [51].

Duru et al.’s investigation involved evaluating how well isoflavones (daidzein, genistein, puerarin) are extracted from the by-products of Kudzu roots using natural deep eutectic solvents coupled with ultrasound-supported extraction. The use of natural deep eutectic solvents coupled with ultrasound-assisted extraction was evaluated against the usual Soxhlet extraction technique, and the amounts of the extracted isoflavones were determined by HPLC-UV/VIS. The results of this study suggest that the developed technique may have advantages such as reduced extraction time and the use of inexpensive and green extraction solvents, but further investigation is needed to fully optimize the conditions for extracting isoflavones [52]. Below, the most commonly used extraction techniques are presented with different advantages and disadvantages (Table 1).

In summary, different extraction methods, including ultrasound-assisted extraction, supercritical fluid extraction, enzyme-assisted extraction, microwave-assisted extraction, and deep eutectic solvents extraction, provide distinct advantages in the extraction of puerarin from Kudzu root or other plants of the genus *Pueraria* [46]. Various factors affect the extraction of plant sources, including the type of plant material, solvent selection, extraction technique, and operating conditions [47,68].

Understanding these key factors is necessary to optimize the extraction process and achieve high yields with desired properties. The primary variable in any extraction method is definitely the solvent selection. It is important to choose the extraction solvent based on its solubility and the intensity of interactions with the matrix. To examine the solvent’s properties, it is necessary to pay attention to polarity, pH, viscosity, surface tension, vapor pressure, boiling point, solid–liquid ratio, as well as the effect on the purity and activity of the extracted compound [68].

For example, in the case of ultrasound-assisted extraction, at the adjusted temperature, a solvent with low vapor pressure facilitates cavitation, which increases the impact of ultrasound on the process, and on the other hand viscous solutions have the opposite effect, increasing the amplitude of waves, hindering the propagation of ultrasound, and producing mechanical effects on the sample due to cavitation [68,69]. The frequency of extraction is a crucial parameter that prevents the cavitation process from fully occurring and decreases the size of bubbles by decreasing their expansion time [69]. The dielectric constant and the dissipation factor are crucial parameters for microwave-assisted extraction, and modifying the dielectric constant is necessary to obtain suitable characteristics. More microwave energy is required for high volumes of solvent because microwave radiation is absorbed by the solvent [68,70]. Very high microwave power can lead to lower yields, which can be attributed to the heat generated by the microwave energy causing the disintegration and thermal degradation of the puerarin content.

The potential of these advanced techniques in improving extraction efficiency, reducing processing time, and enhancing the quality of extracted puerarin is significant. Additionally, these extraction methods can also integrate green solvents and environmentally friendly approaches, which promote sustainability and align with the industry’s growing demand for chemical-free and eco-friendly processes, according to the basics and fundamentals of green chemistry [71,72].

Analytical techniques are crucial for the pharmaceutical industry, as they aid in comprehending the physical and chemical stability of the bioactive compound, which influences the selection and design of the dosage form, assesses stability, and identifies the impurities [73]. To determine the presence and concentration of puerarin, various analytical methods can be employed. Spectroscopic methods such as UV-VIS spectrophotometry and mass spectrometry are included, along with chromatographic techniques such as high-performance liquid chromatography and gas chromatography [74,75].

One of the commonly used methods for the analysis of puerarin is high-performance liquid chromatography (HPLC) [76,77,78]. HPLC is a popular technique for estimating puerarin concentration because it allows for the separation and quantification of individual components in a sample. Choosing an analytical method for puerarin determination necessitates considering factors like sensitivity, selectivity, and reproducibility. The accuracy and precision of the results can only be guaranteed by verifying the chosen method through standardization and calibration [76,79].

Even though high-performance liquid chromatography and mass spectrometry are widely used for analyzing puerarin, it is essential to critically evaluate the limitations and potential drawbacks that arise with these approaches. Depending on the cost of the equipment and the specialized training needed for the operation, accessibility can be limited [79]. In addition to the analytical methods mentioned, nuclear magnetic resonance (NMR) spectroscopy is another effective method for analyzing puerarin. The identification and quantification of puerarin in complex samples, such as plant extracts, can be achieved through NMR spectroscopy, which provides detailed information about the molecular structure and dynamics of compounds. For example, Yi et al. performed a complete NMR analysis of puerarin and explored the antioxidative activity by bond dissociation enthalpy (BDE) calculations. Their results revealed that in methanol-d4, the PBE0/aug-cc-pVTZ approach was employed to calculate the root mean square value of puerarin to 5.73 ppm. Also, they calculated the ^13^C and ^1^H chemical shifts of the puerarin molecule (in C7 and C4’ positions) in methanol-d4, phenolic O–H bond dissociation enthalpies (84.3 kcal·mol^−1^; 82.5 kcal·mol^−1^), and single-step hydrogen atom transfer [80].

Integrating NMR analysis with other analytical techniques (e.g., HPLC, MS) can lead to a more comprehensive assessment of bioactive molecules, which allows researchers to take advantage of the strengths of both methods while minimizing their limitations [81,82,83]. Furthermore, these analytical methods offer precise information about the molecular structure of puerarin, which makes it easier to identify and quantify complex samples (Table 2).

## 4. Biological Effects of Puerarin

Chronic diseases are becoming more prevalent as a result of the increasing ill population worldwide, leading to a serious threat to the health of individuals. Although new drugs are being developed to improve health, there has been insufficient progress in this area.

Plant-derived natural preparations have become a valuable resource for the development of new drugs. Bioactive molecules from Chinese herbal medicines (e.g., ginseng, astragalus, Ginkgo biloba) have been discovered to have ‘life-nourishing’ properties, and their role in health is being more and more recognized [91,92,93].

Puerarin has also gained recognition over the years due to its diverse pharmacological and biological effects in the treatment of acute and chronic diseases [28,94,95,96,97,98], such as cardiovascular diseases [99,100,101,102], liver diseases [103,104,105], neurologic disorders [26,106,107], respiratory diseases [108], and many more.

The structure–activity relationship of puerarin has been the subject of numerous studies conducted by researchers in recent decades. Analyzing the relationship between the structure and activity of puerarin allows us to develop more effective analogues that can highlight more pronounced biological effects, particularly in oncological drug development. Discovering new biological effects is also a focus of current research. Current research also focuses on the discovery of new biological effects. The development of an active pharmaceutical form in which puerarin is encapsulated has been the focus of fewer clinical studies. Besides discovering new biological effects, it is also essential to monitor the potential adverse effects that may occur.

In the forthcoming section, we summarize an analysis of the biological effects of puerarin (Table 3).

## 5. Future Perspectives

*Pueraria* species have been employed in China to treat a range of illnesses for thousands of years. Numerous impressive achievements have been made and more studies have been conducted in recent decades. Biotechnology has led to the development of new extraction methods that can extract and isolate more biologically active components from medicinal plants, which has resulted in the introduction of drugs into clinics or supplements for the pharmaceutical market.

Puerarin, which is an active ingredient in traditional herbal medicine, has been acknowledged to possess a variety of biological effects. Numerous studies are beginning to find solutions to the issues that require puerarin to be used as a therapeutic agent, such as its limited bioavailability caused by its low solubility and lipid stability.

It is undeniable that biotechnology is a tool for achieving sustainable processes and products. The specificity, activity, and stability of enzymes are expected to be expanded in green chemistry and biotechnology as a result of advances in enzyme engineering and biocatalyst optimization. The demand for eco-friendly and cost-effective synthetic routes, particularly for addressing puerarin issues, will make enzymatic synthesis a key factor in innovation and progress in the years ahead [141]. Furthermore, enzymatic synthesis will be enhanced by applying advanced computational tools and machine learning algorithms to design and optimize enzymes for specific synthesis pathways for different drugs or natural compounds, such as puerarin [142,143].

As the field of enzymatic synthesis progresses, there are several emerging trends that could revolutionize the production and use of nanoparticles. Metal nanoparticles, solid lipid nanoparticles, nanomicelles, cyclodextrins, dendrimers, and nano-vesicle systems are among the most common nanoparticles that have been studied for their biocompatibility and biodegradability [144,145,146]. The encapsulation of various nanoparticles with puerarin has been carried out by researchers for many years to improve its bioavailability and therapeutic effects, and the results have been promising [147,148,149,150]. Enzymatic synthesis and nanoparticle production have immense potential due to ongoing research and innovation, which will lead to more efficient, sustainable, and versatile manufacturing processes.

This review provides a preliminary up-to-date overview of puerarin’s biosynthesis, extraction methods, analytical techniques, and bioactivities, with emphasis on its potential as a bioactive molecule in the treatment of various systemic diseases. As this field’s research progresses, it is evident that the development of innovative extraction techniques will have a significant impact on improving the production and utilization of puerarin in pharmaceuticals, food supplements, and other related products. With more in-depth experimental and clinical studies on puerarin, its biological activity mechanism will be more fully revealed, the types of medication will be more varied, and the clinical indications will be expanded in the future.

## Figures and Tables

**Table 1:** Advantages and disadvantages of various techniques for extracting puerarin from natural sources.

Extraction Techniques	Advantages	Disadvantages	References
Traditional (e.g., Maceration, Percolation, Decoction, Soxhlet)	▪ Low installation cost ▪ Low maintenance cost ▪ Dynamic extraction	Time-consuming process Poor purity Low efficiency Large amounts of potentially toxic solvents Significant waste production Difficulty with automation	[47,48,53]
Microwave-Assisted Extraction	▪ Shorter time ▪ Higher extraction rate ▪ Low consumption of organic solvents ▪ Lower costs ▪ Low pollution	Generating free radicals Heating occurs during extraction Restricted to polar solvents Not specifically for volatile solvents	[46,47,48,53,54,55,56]
Ultrasound-Assisted Extraction	▪ Heating is not necessary ▪ High efficiency and yields ▪ Low energy consumption ▪ Less solvent	Excessive ultrasonic energy may lead to degradation of puerarin	[47,48,53,57,58,59,60]
Supercritical Fluid Extraction	▪ Environmentally friendly ▪ High selectivity ▪ Mild extraction conditions ▪ Requires less energy and resources	Limited mass transfer Expensive equipment Required technical knowledge of different specific properties	[46,47,48,61,62]
Enzyme-Assisted Extraction	▪ Gentle reaction conditions ▪ Eco-friendly extraction solvents ▪ Minimal active substance loss ▪ Mild conditions ▪ Higher extraction rate ▪ Possibility of combining with various extraction methods (e.g., ultrasound-assisted extraction, microwave-assisted extraction)	High cost of enzymes	[46,47,48,63]
Deep Eutectic Solvents Extraction	▪ Green solvents ▪ DES are extremely easy to prepare with high purity ▪ Lower extraction temperature ▪ Lower costs ▪ Shorter extraction time ▪ High selectivity	Limited mass transfer High density and viscosity Low vapor pressure	[64,65,66,67]

**Table 2:** Details of various analytical methods used for the estimation of puerarin.

Analyte	Column; Mobile Phase	Flow Rate; Temperature; Detection Wavelength	Combined Technique Parameters	Results	References
Puerarin (*Pueraria lobata* stem extract, puerarin cream)	Optimapark C18 column (250 × 4.6 mm, 5 μm); A: 0.5% aqueous acetic acid; B: methanol (77:23, *v*/*v*)	1 mL/min; 30 °C; 250 nm	-	Retention time = 10.75 min; Total analysis time = 25 min; Puerarin content in extract (0.29 ± 0.01%); puerarin content cream (0.015 ± 0.001%); This analytical method was successfully applied to quality control of raw material and cosmetic product.	[84]
Puerarin (*Pueraria lobata*)	Optimapark C18 column (4.6 mm × 250 mm, 5 μm); A: 0.1% formic acid/aqueous solution; B: acetonitrile	1 mL/min	PDA–ESI–MS/MS: ○ Detection: 200 ÷ 400 nm; ○ Drying gas flow: 15 L/min; ○ Nebulizing gas flow rate: 3 L/min; ○ Desolvation line temperature: 250 °C; ○ Heat block temperature: 400 °C.	Retention time = 15.43 min; [M+H]^+^ = 417.10; [M−H]^−^ = 415.09; Product ion (*m*/*z*) = 297.12; λ Max (nm) = 250, 305.	[85]
Puerarin (*Pueraria lobata* radix)	ZORBAX SB C18 reversed-phase column (4.6 mm × 250 mm, 5 μm); A: 0.2% phosphoric acid/ water; B: methanol	1 mL/min; 35 °C; 475 nm	-	Precision (RSD) = 0.40–1.63%; Stability (RSD) = 1.05–4.95%; Repeatability (RSD) = 2.52–4.95%; LODs = 0.0152–0.0307 μg/mL; LOQs = 0.0506–0.1024 μg/mL; The maximum extraction efficiency reached 8.92 mg/g with 7.66 mg/g puerarin.	[86]
Puerarin (*Pueraria lobata*)	Agilent SB-C18 (2.1 mm × 100 mm, 1.8 μm); A: water/0.1% formic acid; B: acetonitrile/0.1% formic acid	40 °C	ESI–(QTRAP)–MS: ○ Source temperature: 550 °C; ○ Ion spray voltage (IS): 5500 V (positive ion mode)/−4500 V (negative ion mode); ○ Source: gas I, gas II, and curtain gas (50, 60, and 25.0 psi).	Retention time = 3.22 min; [M+H]^+^ = 417.	[87]
Puerarin (gel eye drops)	Agilent Zorbax SB-C18 column (3.0 × 150 mm, 3.5 μm); A: acetonitrile gradient; B: 0.1% formic acid (15:85, v:v)	0.6 mL/min; 35 °C; 250 nm	MS: ○ Nebulizing gas: 55 psi; ○ Turbo ion spray ○ temperature = 600 °C; ○ Collision gas = 8 psi; ○ Curtain gas = 20 psi; ○ Ion spray ○ voltage: −4500 V.	Product ion (*m*/*z*) = 415.1; The declustering potential is −80 V, entrance potential is −10 V, collision energy is −45 V, and collision cell exit potential is −10 V; Three levels of quality control samples (LQC 6 ng/mL, MQC 150 ng/mL, and HQC 3200 ng/mL) as well as LLOQ (2 ng/mL); The RSDs were below 10% for intra- and inter-day precision measurements, and the accuracy ranged from 92.3 to 104.0%, suggesting their analytical approach was reliable and acceptable for quantifying PUE in aqueous humor.	[88]
Puerarin (*Pueraria* *thomsonii* radix)	Waters BEH C18 column (2.1 mm × 100 mm, 1.7 μm); A: 0.1% formic acid/water; B: 0.1% formic acid/acetonitrile	0.3 mL/min; 30 °C	Q-TOF-MS: ○ Range (*m*/*z*): 100–2000; ○ Source voltages of 5500 V (positive ion) and −4500 V (negative ion); ○ Ion source temperatures = 600 °C and 500 °C; ○ Cracking voltage (±80 V); ○ Collision energy (±10 eV).	Selected ion: [M−H]^−^; Retention time = 5.14 min; Calculated and measured mass = 415.1036; RSD = 0.73%; Reproducibility (RSD) = 0.15%; The extract contained 2.1145 mg/mL puerarin.	[89]
Puerarin (*Pueraria* *tuberosa*)	C18 (250 mm × 4.6 mm); A: methanol; B: water (25:27 ratio)	1 mL/min; 25 °C; 250 nm	Q-TOF-MS	Retention time = 18.156 min; [M+H]^+^ = 417.1201; [M+Na]^+^ = 439.1015.	[90]

**Table 3:** Summary of the various biological effects of puerarin.

Type of Disease/Disorder	Biological Effects of Puerarin	References
Cardiovascular disease	▪ Inhibits or regulates critical molecular activities involved in the major cellular events of cardiac remodeling, such as JNK1/2, AMPK/mTOR, PPAR α/γ, Na^+^/K^+^-ATPase, HIF-1 α, angiopoietin, and myocardial death-related pathways (e.g., mitochondrial apoptosis, necrosis, autophagy); ▪ Relieves the effects of oxidative stress and inflammation; ▪ Improves mitochondrial function; ▪ Decreases the death of cardiomyocytes.	[109]
	▪ Alleviates hyperpermeability by decreasing the levels of TNF-α and IL-1β; ▪ Inhibits the expression of adhesion molecules, and the inflammatory factors IL-8, COX-2, IL-1β, TNF-α, and IL-6 to exert an anti-inflammatory effect in atherosclerosis; ▪ Improves the lipid profile by reducing the levels of blood triglyceride, total cholesterol, and low-density lipoprotein cholesterol; ▪ Increases the levels of high-density lipoprotein cholesterol in hyperlipidemic rats; ▪ Improves regulation of Na^+^/K^+^-ATPase; ▪ Decreases inflammation, oxidative stress, autophagy, and myocardial fibrosis.	[110]
	▪ Reduces myocardial remodeling-related proteins expression; ▪ Attenuates reactive oxygen species, restores mitochondrial membrane potential, and decreases Ca^2+^-overload *in vitro*.	[111]
	▪ Anti-myocardial fibrosis and anti-myocardial ischemia effects; ▪ Inhibits myocardial hypertrophy; ▪ Anti-atherosclerosis effects; ▪ Inhibits the activation of p38-MAPK and reduces the content of TNF-a in serum; ▪ Inhibits the activity of myeloperoxidase and decreases malondialdehyde content in the myocardial tissue; ▪ Lowers blood pressure and enhances vascular endothelial function by relaxing blood vessels through the eNOS signaling pathway.	[112]
	▪ Inhibits excess oxidative stress and the release of inflammatory cytokines; ▪ Maintains mitochondrial function; ▪ Promotes adaptive autophagy and protects against myocardial damage.	[113]
	▪ Puerarin pretreatment reduces the cardiotoxicity injury associated with doxorubicin, resulting in increased cell viability, decreased LDH activity, and apoptosis; ▪ Prevents excess oxidative stress, maintains mitochondrial function and energy metabolism, and enhances myocardial function.	[114]
	▪ The reduction of IL-1β was positively correlated with succinate in the serum of puerarin–tanshinone IIA-treated mice; ▪ Inhibits inflammation by targeting HIF-1α to interfere with the succinate signaling axis; ▪ The combination of puerarin–tanshinone IIA was more effective in restraining inflammatory responses and the formation of atherosclerotic plaque.	[115]
Liver disease	▪ Hepatoprotective effects against benzo[a]pyrene-induced liver damage via inhibiting oxidative stress and inflammation.	[116]
	▪ Inhibits mPTP opening, and decreases mitochondrial Ca^2+^ levels and ATP synthase expression; ▪ Corrects the pathological damage caused by *Xanthium strumarium* toxicity.	[117]
	▪ Attenuates EtOH-induced liver injury; ▪ Inhibits levels of SREBP-1c, TNF-α, IL-6 and IL-1β, compared with silymarin; ▪ Acts as an inhibitor of MMP8 to reduce inflammation and lipid deposition in alcoholic-liver disease.	[118]
Respiratory disease	▪ Inhibits the inflammatory response to prevent LPS-induced acute lung injury; ▪ Reduces LPS-induced damage to A549 cells; ▪ Reduces the expression of the inflammatory cytokines TNF-α, IL-8, and IL-1β in LPS-induced A549 cells; ▪ Improves sepsis-induced lung injury by inhibiting ferroptosis.	[119]
	▪ Redox-sensitive attenuation effect of inflammatory responses in mice exposed to ACS- and CSE-stimulated HSAECs, via inhibition of NOX-isoforms; ▪ Reduces the production of reactive oxygen species, lowers the infiltration of inflammatory cells, and decreases the expression of inflammatory mediators.	[120]
Gastric disease	▪ Decreases NLRP3 inflammasome-mediated injury by inducing AMPK/SIRT1 signaling in the gastric epithelium; ▪ Protects GES-1 cells against LPS-induced injury by inhibiting NLRP3.	[121]
Kidney disease	▪ Reduces p65 acetylation *via* Sirt1 activation; ▪ Additive inhibitory effects on the NF-κB activation.	[122]
	▪ Improves glucose level and lipid metabolism; ▪ Suppresses the production of reactive oxygen species and the accumulation of excessive collagen fiber in glomerular mesangial cells; ▪ Downregulates TGF-β and mesenchymal transition markers in high glucose-injured glomerular mesangial cells and diabetic kidney.	[123]
Metabolic disease	▪ The synthesis of the chitosan–puerarin hydrogel led to the discovery that it promotes diabetic wound healing by inhibiting ectopic miR-29ab1-mediated macrophages and controlling inflammation.	[124]
Neurological disorders	▪ Significantly attenuates depression-like behaviors in rats; ▪ Controls the imbalance of intestinal bacteria; ▪ Inhibits inflammatory responses in the hippocampus, serum, colon, and downregulates the TLR4/NF-κB pathway.	[125]
	▪ Improves neurological impairment and forelimb motor function; ▪ Reduces inflammatory response; ▪ Inhibits brain edema; ▪ Regulates synaptic plasticity.	[126]
	▪ Induces brain-derived neurotrophic factor production in astrocytes; ▪ Promotes phosphorylation of extracellular-signal-regulated kinases; ▪ Protects astrocytes through the PI3K/Akt- and ERK/mitogen-activated protein kinases pathway; ▪ Increases the potential of the mitochondrial membrane; ▪ Decreases mitochondrial reactive oxygen species; ▪ Increases Bcl-2 and decreases Bax levels; ▪ Suppresses caspase-3 activation; ▪ Decreases the production of pro-inflammatory cytokines; ▪ Inhibits inflammatory responses; ▪ Downregulates apoptosis-associated proteins; ▪ Reduces calcium influx.	[127]
	▪ Significantly reduces the production of inflammatory cytokines (e.g., TNF-α, IL-6) in the peripheral blood; ▪ Exhibits inhibitory effects on the release of TNF-α and IL-6 from microglia, preventing hippocampal neuronal cell death; ▪ Exerts anti-neuroinflammatory effect against sepsis-associated encephalopathy by modulating the AKT1 pathway in microglia.	[128]
	▪ Inhibits the level of factors related to the classical pathway of pyroptosis (e.g., NLRP3, Caspase-1, IL-1β, IL-18); ▪ Reduces blood–brain barrier damage.	[129]
	▪ Attenuates oxidative stress and neuron apoptosis; ▪ Enhances synaptic plasticity; ▪ Improves cognitive function by blocking the TRPM2/NMDA receptor pathway; ▪ Inhibits oxidative stress, apoptosis, and autophagy deficits by promoting synaptic plasticity and suppressing oxidative stress, apoptosis, and autophagy deficits.	[130]
Urologic disease	▪ Induces proliferation inhibition, apoptosis, and senescence of bladder cancer cells *in vitro*; ▪ Inhibits CCNB1 and PI3K/AKT pathways by upregulating the miR-139-5p; ▪ Exerts oncogenic effects in bladder cancer by regulating the miR-139-5p/CCNB1 and PI3K/AKT pathways.	[131]
Geriatric disease	▪ Reduces bone loss; ▪ Increases bone density; ▪ Boosts osteogenic activity; ▪ Helps promote bone repair and remodeling, which can be beneficial after bone transplantation or in patients with osteoporosis; ▪ Exerts inhibitory effects on the adipogenic differentiation of bone marrow mesenchymal stem cells; ▪ Prevents alcoholic osteonecrosis;	[132]
	▪ Encapsulation of puerarin into peptide self-assembled hydrogels significantly ameliorates the progression of monoiodoacetic acid-induced osteoarthritis in rats.	[133]
Bone disease	▪ Significantly inhibits osteoclast activation and bone resorption, without affecting osteoclastogenesis or apoptosis; ▪ Significantly blocks c-Fos expression.	[134]
	▪ Anti-osteoporosis effect; ▪ Reduces adipogenic differentiation and promotes osteogenic differentiation of bone mesenchymal stem cells via activating the Wnt/β-catenin pathway and inhibiting the PPARγ pathway.	[135]
	▪ Inhibits the activity and differentiation of osteoclasts; ▪ Inhibits osteoclast differentiation through the OPG/RANK/RANKL signaling pathway.	[136]
Ophthalmology disease	▪ Inhibits amyloid β-induced NLRP3 inflammasome activation in retinal pigment epithelial cells *via* suppressing ROS-dependent oxidative and endoplasmic reticulum stresses; ▪ Promotes the activity of superoxide dismutase, catalase, and glutathione; ▪ Inhibits the expression of nNOS and MDA to protect against retinal damage caused by oxidative stress; ▪ Improves micro-circulation; ▪ Reduces blood viscosity; ▪ Improves the reduction of intraocular pressure.	[137]
	▪ Treatment with puerarin enhances cell viability, reduces reactive oxygen species content, increases catalase and superoxide dismutase activities, and elevates the ratio of GSH/GSSG in human corneal epithelial cells; ▪ Attenuates hyperosmotic stress-induced injury of the human corneal epithelial cell line by targeting the regulation of the SIRT1/NLRP3 signaling.	[138]
Sensorial disorders	▪ Anti-apoptotic effects towards ototoxic drug (e.g., cisplatin)-induced hair cell injury *in vitro*; ▪ Suppression of the synthesis of reactive oxygen species; ▪ Inhibits apoptosis, and upregulates the Akt signaling pathway.	[139]
Pregnancy-specific disorder	▪ Protection against H_2_O_2_-induced apoptosis in HTR-8/SVneo cells by regulating the miR-20a-5p/VEGFA/Akt axis; ▪ Reverses H_2_O_2_-induced apoptosis and metastasis inhibition in cells; ▪ Provides some theoretical basis for exploring effective treatments for patients with preeclampsia.	[140]

## Data Availability

Not applicable.

## References

[1] Moses T., Goossens A. (2017). Plants for Human Health: Greening Biotechnology and Synthetic Biology. J. Exp. Bot..

[2] Schaal B. (2019). Plants and People: Our Shared History and Future. Plants People Planet.

[3] Chaachouay N., Zidane L. (2024). Plant-Derived Natural Products: A Source for Drug Discovery and Development. Drugs Drug Candidates.

[4] Pergola M., De Falco E., Belliggiano A., Ievoli C. (2024). The Most Relevant Socio-Economic Aspects of Medicinal and Aromatic Plants through a Literature Review. Agriculture.

[5] Carrubba A., Marceddu R., Sarno M. Bringing Spontaneous Plants to Cultivation: Issues and Constraints for Medicinal and Aromatic Plants. Proceedings of the XXXI International Horticultural Congress (IHC2022): International Symposium on Medicinal and Aromatic Plants: Domestication, Breeding, Cultivation and New Perspectives.

[6] Ansari M.K.A., Iqbal M., Chaachouay N., Ansari A.A., Owens G. (2023). The Concept and Status of Medicinal and Aromatic Plants: History, Pharmacognosy, Ecology, and Conservation. Plants as Medicine and Aromatics.

[7] Azaizeh H., Saad B., Cooper E., Said O. (2010). Traditional Arabic and Islamic Medicine, a Re-Emerging Health Aid. Evid.-Based Complement. Altern. Med..

[8] Hamilton A.C. (2004). Medicinal Plants, Conservation and Livelihoods. Biodivers. Conserv..

[9] Woo S., Marquez L., Crandall W.J., Risener C.J., Quave C.L. (2023). Recent Advances in the Discovery of Plant-Derived Antimicrobial Natural Products to Combat Antimicrobial Resistant Pathogens: Insights from 2018–2022. Nat. Prod. Rep..

[10] Yu J., Zheng Y., Song C., Chen S. New Insights into the Roles of Fungi and Bacteria in the Development of Medicinal Plant. J. Adv. Res..

[11] Hui Z., Wen H., Zhu J., Deng H., Jiang X., Ye X.Y., Wang L., Xie T., Bai R. (2024). Discovery of Plant-Derived Anti-Tumor Natural Products: Potential Leads for Anti-Tumor Drug Discovery. Bioorg Chem..

[12] Peterle L., Sanfilippo S., Borgia F., Li Pomi F., Vadalà R., Costa R., Cicero N., Gangemi S. (2023). The Role of Nutraceuticals and Functional Foods in Skin Cancer: Mechanisms and Therapeutic Potential. Foods.

[13] Phillipson J.D. (2007). Phytochemistry and Pharmacognosy. Phytochemistry.

[14] Teoh E.S. (2016). Secondary Metabolites of Plants. Medicinal Orchids of Asia.

[15] Zhao J.H., Wang Y.W., Yang J., Tong Z.J., Wu J.Z., Wang Y.B., Wang Q.X., Li Q.Q., Yu Y.C., Leng X.J. (2023). Natural Products as Potential Lead Compounds to Develop New Antiviral Drugs over the Past Decade. Eur. J. Med. Chem..

[16] Chen X., Lan W., Xie J. (2024). Natural Phenolic Compounds: Antimicrobial Properties, Antimicrobial Mechanisms, and Potential Utilization in the Preservation of Aquatic Products. Food Chem..

[17] Liga S., Paul C., Péter F. (2023). Flavonoids: Overview of Biosynthesis, Biological Activity, and Current Extraction Techniques. Plants.

[18] Lv J., Yang S., Zhou W., Liu Z., Tan J., Wei M. (2024). Microbial Regulation of Plant Secondary Metabolites: Impact, Mechanisms and Prospects. Microbiol. Res..

[19] Su Y., Wang J., Gao W., Wang R., Yang W., Zhang H., Huang L., Guo L. (2023). Dynamic Metabolites: A Bridge between Plants and Microbes. Sci. Total Environ..

[20] Wrońska N., Szlaur M., Zawadzka K., Lisowska K. (2022). The Synergistic Effect of Triterpenoids and Flavonoids—New Approaches for Treating Bacterial Infections?. Molecules.

[21] Pietta P., Minoggio M., Bramati L., Rahman A. (2003). Plant Polyphenols: Structure, Occurrence and Bioactivity. Studies in Natural Products Chemistry.

[22] Chen S., Wang X., Cheng Y., Gao H., Chen X. (2023). A Review of Classification, Biosynthesis, Biological Activities and Potential Applications of Flavonoids. Molecules.

[23] Alexander V.S. (2014). Phytoestrogens and Their Effects. Eur. J. Pharmacol..

[24] Křížová L., Dadáková K., Kašparovská J., Kašparovský T. (2019). Isoflavones. Molecules.

[25] Tan S.T., Tan S.S., Tan C.X. (2023). Soy Protein, Bioactive Peptides, and Isoflavones: A Review of Their Safety and Health Benefits. PharmaNutrition.

[26] Ren Y., Qu S. (2023). Constituent Isoflavones of *Puerariae radix* as a Potential Neuroprotector in Cognitive Impairment: Evidence from Preclinical Studies. Ageing Res. Rev..

[27] Gómez-Zorita S., González-Arceo M., Fernández-Quintela A., Eseberri I., Trepiana J., Portillo M.P. (2020). Scientific Evidence Supporting the Beneficial Effects of Isoflavones on Human Health. Nutrients.

[28] Zhou Y.-X., Zhang H., Peng C. (2014). Puerarin: A Review of Pharmacological Effects. Phytother. Res..

[29] Esch H.L., Kleider C., Scheffler A., Lehmann L., Gupta R.C. (2016). Chapter 34—Isoflavones: Toxicological Aspects and Efficacy. Nutraceuticals.

[30] Bacanlı M., Aydın S., Başaran A.A., Başaran N., Watson R.R., Preedy V.R., Zibadi S. (2018). Chapter 33—A Phytoestrogen Puerarin and Its Health Effects. Polyphenols: Prevention and Treatment of Human Disease.

[31] Dias M.C., Pinto D.C.G.A., Silva A.M.S. (2021). Plant Flavonoids: Chemical Characteristics and Biological Activity. Molecules.

[32] Tariq H., Asif S., Andleeb A., Hano C., Abbasi B.H. (2023). Flavonoid Production: Current Trends in Plant Metabolic Engineering and De Novo Microbial Production. Metabolites.

[33] Han R., Takahashi H., Nakamura M., Yoshimoto N., Suzuki H., Shibata D., Yamazaki M., Saito K. (2015). Transcriptomic Landscape of Pueraria Lobata Demonstrates Potential for Phytochemical Study. Front. Plant Sci..

[34] Wang X., Li C., Zhou C., Li J., Zhang Y. (2017). Molecular Characterization of the C-Glucosylation for Puerarin Biosynthesis in Pueraria Lobata. Plant J..

[35] Cheng H., Huang X., Wu S., Wang S., Rao S., Li L., Cheng S., Li L. (2022). Chromosome-Level Genome Assembly and Multi-Omics Dataset Provide Insights into Isoflavone and Puerarin Biosynthesis in Pueraria Lobata (Wild.) Ohwi. Biomolecules.

[36] Xi H., Zhu Y., Sun W., Tang N., Xu Z., Shang X., Zhang Y., Yan H., Li C. (2023). Comparative Transcriptome Analysis of Pueraria Lobata Provides Candidate Genes Involved in Puerarin Biosynthesis and Its Regulation. Biomolecules.

[37] Hu X., Zhu T., Min X., He J., Hou C., Liu X. (2023). Integrated Metabolomic and Transcriptomic Analysis of Puerarin Biosynthesis in Pueraria Montana Var. Thomsonii at Different Growth Stages. Genes.

[38] Maciejewska-Turska M., Sieniawska E., Xiao J. (2023). Puerarin: Advances on Resources, Biosynthesis Pathway, Bioavailability, Bioactivity, and Pharmacology. Handbook of Dietary Flavonoids.

[39] Li C., Zhang Y. (2023). Glycosylation and Methylation in the Biosynthesis of Isoflavonoids in Pueraria Lobata. Front. Plant Sci..

[40] Adolfo L.M., Burks D., Rao X., Alvarez-Hernandez A., Dixon R.A. (2022). Evaluation of Pathways to the C-Glycosyl Isoflavone Puerarin in Roots of Kudzu (Pueraria Montana Lobata). Plant Direct.

[41] Tungmunnithum D., Intharuksa A., Sasaki Y. (2020). A Promising View of Kudzu Plant, Pueraria Montana Var. Lobata (Willd.) Sanjappa & Pradeep: Flavonoid Phytochemical Compounds, Taxonomic Data, Traditional Uses and Potential Biological Activities for Future Cosmetic Application. Cosmetics.

[42] Kato-Noguchi H. (2023). The Impact and Invasive Mechanisms of Pueraria Montana Var. Lobata, One of the World’s Worst Alien Species. Plants.

[43] Chen K., Wei P., Jia M., Wang L., Li Z., Zhang Z., Liu Y., Shi L. (2024). Research Progress in Modifications, Bioactivities, and Applications of Medicine and Food Homologous Plant Starch. Foods.

[44] Bharti R., Chopra B.S., Raut S., Khatri N. (2021). *Pueraria tuberosa*: A Review on Traditional Uses, Pharmacology, and Phytochemistry. Front. Pharmacol..

[45] Fu M., Jahan M.S., Tang K., Jiang S., Guo J., Luo S., Luo W., Li G. (2023). Comparative Analysis of the Medicinal and Nutritional Components of Different Varieties of Pueraria Thomsonii and Pueraria Lobata. Front. Plant Sci..

[46] Xuan T., Liu Y., Liu R., Liu S., Han J., Bai X., Wu J., Fan R. (2023). Advances in Extraction, Purification, and Analysis Techniques of the Main Components of Kudzu Root: A Comprehensive Review. Molecules.

[47] Tzanova M., Atanasov V., Yaneva Z., Ivanova D., Dinev T. (2020). Selectivity of Current Extraction Techniques for Flavonoids from Plant Materials. Processes.

[48] Chávez-González M.L., Sepúlveda L., Verma D.K., Luna-García H.A., Rodríguez-Durán L.V., Ilina A., Aguilar C.N. (2020). Conventional and Emerging Extraction Processes of Flavonoids. Processes.

[49] Abhari K., Mousavi Khaneghah A. (2020). Alternative Extraction Techniques to Obtain, Isolate and Purify Proteins and Bioactive from Aquaculture and by-Products. Advances in Food and Nutrition Research.

[50] Bagade S.B., Patil M. (2021). Recent Advances in Microwave Assisted Extraction of Bioactive Compounds from Complex Herbal Samples: A Review. Crit. Rev. Anal. Chem..

[51] Zhu H., Xing Y., Akan O.D., Yang T. (2023). Ultrafine Comminution-Assisted Ultrasonic-Microwave Synergistic Extraction of Pueraria Mirifica (Kudzu Flower and Root) Flavonoids. Heliyon.

[52] Duru K.C., Slesarev G.P., Aboushanab S.A., Kovalev I.S., Zeidler D.M., Kovaleva E.G., Bhat R. (2022). An Eco-Friendly Approach to Enhance the Extraction and Recovery Efficiency of Isoflavones from Kudzu Roots and Soy Molasses Wastes Using Ultrasound-Assisted Extraction with Natural Deep Eutectic Solvents (NADES). Ind. Crops Prod..

[53] Blicharski T., Oniszczuk A. (2017). Extraction Methods for the Isolation of Isoflavonoids from Plant Material. Open Chem..

[54] Zhang Y., Han L., Zou L., Zhang M., Chi R. (2021). Development of an SVR Model for Microwave-Assisted Aqueous Two-Phase Extraction of Isoflavonoids from *Radix Puerariae*. Chem. Eng. Commun..

[55] Liu Y.-K., Yan E., Zhan H.-Y., Zhang Z.-Q. (2011). Response Surface Optimization of Microwave-Assisted Extraction for HPLC-Fluorescence Determination of Puerarin and Daidzein in *Radix Puerariae thomsonii*. J. Pharm. Anal..

[56] Nour A.H., Oluwaseun A.R., Nour A.H., Omer M.S., Ahmed N., Churyumov G.I. (2021). Microwave-Assisted Extraction of Bioactive Compounds (Review). Microwave Heating.

[57] Zou Y., Tian M., Liu C. (2016). Optimization of Ultrasound-Assisted Extraction of Puerarin from Pueraria Lobata Dried Root. J. Food Process Preserv..

[58] Zheng X., Chen J., Jiang X., Guo Q., Deng Z., Li H. (2015). Ultrasonic-Assisted Extraction of Puerarin Optimized by Response Surface Methodology. Proceedings of the 2015 Chinese Intelligent Automation Conference.

[59] Aihua S., Xiaoyan C., Xiaoguang Y., Jiang F., Yanmin L., Zhou J. (2019). Applications and Prospects of Ultrasound-Assisted Extraction in Chinese Herbal Medicine. Open Access J. Biomed. Sci..

[60] Zeng X., Tan H., Liu B., Wen Y. (2023). Optimization of Ultrasonic-Assisted Extraction and Purification of Total Flavonoids with Biological Activities from *Radix Puerariae*. Biomass Convers. Biorefin.

[61] Vinitha U.G., Sathasivam R., Muthuraman M.S., Park S.U. (2022). Intensification of Supercritical Fluid in the Extraction of Flavonoids: A Comprehensive Review. Physiol. Mol. Plant Pathol..

[62] Khaw K.Y., Parat M.O., Shaw P.N., Falconer J.R. (2017). Solvent Supercritical Fluid Technologies to Extract Bioactive Compounds from Natural Sources: A Review. Molecules.

[63] Majik M.S., Gawas U.B., Meena S.N., Nandre V., Kodam K., Meena R.S. (2023). Chapter 2—Recent Advances in Extraction of Natural Compounds. New Horizons in Natural Compound Research.

[64] Huang Y., Yang J., Zhao Y., Yu L., He Y., Wan H., Li C. (2021). Screening, Optimization, and Bioavailability Research of Natural Deep Eutectic Solvent Extracts from *Radix Pueraria*. Molecules.

[65] Makkliang F., Siriwarin B., Yusakul G., Phaisan S., Sakdamas A., Chuphol N., Putalun W., Sakamoto S. (2021). Biocompatible Natural Deep Eutectic Solvent-Based Extraction and Cellulolytic Enzyme-Mediated Transformation of Pueraria Mirifica Isoflavones: A Sustainable Approach for Increasing Health-Bioactive Constituents. Bioresour. Bioprocess..

[66] Wang T., Guo Q., Yang H., Gao W., Li P. (2022). PH-Controlled Reversible Deep-Eutectic Solvent Based Enzyme System for Simultaneous Extraction and in-Situ Separation of Isoflavones from Pueraria Lobata. Sep. Purif. Technol..

[67] Kaoui S., Chebli B., Zaidouni S., Basaid K., Mir Y. (2023). Deep Eutectic Solvents as Sustainable Extraction Media for Plants and Food Samples: A Review. Sustain. Chem. Pharm..

[68] Chaves J.O., de Souza M.C., da Silva L.C., Lachos-Perez D., Torres-Mayanga P.C., Machado A.P.d.F., Forster-Carneiro T., Vázquez-Espinosa M., González-de-Peredo A.V., Barbero G.F. (2020). Extraction of Flavonoids From Natural Sources Using Modern Techniques. Front. Chem..

[69] Jurinjak Tušek A., Šamec D., Šalić A. (2022). Modern Techniques for Flavonoid Extraction—To Optimize or Not to Optimize?. Appl. Sci..

[70] Routray W., Orsat V. (2012). Microwave-Assisted Extraction of Flavonoids: A Review. Food Bioprocess Technol..

[71] Ameta S.C., Ameta R. (2023). Green Chemistry: Fundamentals and Applications.

[72] Medina Valderrama C.J., Morales Huamán H.I., Valencia-Arias A., Vasquez Coronado M.H., Cardona-Acevedo S., Delgado-Caramutti J. (2023). Trends in Green Chemistry Research between 2012 and 2022: Current Trends and Research Agenda. Sustainability.

[73] Singh D., Isharani R. (2023). A Detailed Review on Analytical Methods to Manage the Impurities in Drug Substances. OAlib.

[74] Mattrey F.T., Makarov A.A., Regalado E.L., Bernardoni F., Figus M., Hicks M.B., Zheng J., Wang L., Schafer W., Antonucci V. (2017). Current Challenges and Future Prospects in Chromatographic Method Development for Pharmaceutical Research. TrAC-Trends Anal. Chem..

[75] Dispas A., Sacré P.Y., Ziemons E., Hubert P. (2022). Emerging Analytical Techniques for Pharmaceutical Quality Control: Where Are We in 2022?. J. Pharm. Biomed. Anal..

[76] Maji A.K., Banerjee D., Maity N., Banerji P. (2012). A Validated RP-HPLC-UV Method for Quantitative Determination of Puerarin in *Pueraria tuberosa* DC Tuber Extract. Pharm. Methods.

[77] Wu X.-Y., Yang L.-L., Liu-Qing Y., Zou Y.-M., Lu J.-M. (2009). Simultaneous RP-HPLC Determination of Puerarin, Daidzin and Daidzein in Roots, Stems and Leaves of Pueraria Lobata (Wild) Ohwi. Food Sci..

[78] Chauhan S.K., Singh B., Agrawal S. (2004). Determination of Puerarin from Pueraria tuberosa DC by Hplc. Anc. Sci. Life.

[79] Chew Y.L., Khor M.A., Lim Y.Y. (2021). Choices of Chromatographic Methods as Stability Indicating Assays for Pharmaceutical Products: A Review. Heliyon.

[80] Yi Y., Adrjan B., Li J., Hu B., Roszak S. (2019). NMR Studies of Daidzein and Puerarin: Active Anti-Oxidants in Traditional Chinese Medicine. J. Mol. Model..

[81] Shockcor J.P., Lindon J.C., Tranter G.E., Koppenaal D.W. (2017). HPLC–NMR, Pharmaceutical Applications☆. Encyclopedia of Spectroscopy and Spectrometry.

[82] Gebretsadik T., Linert W., Thomas M., Berhanu T., Frew R. (2021). LC–NMR for Natural Product Analysis: A Journey from an Academic Curiosity to a Robust Analytical Tool. Science.

[83] Seger C., Sturm S. (2022). NMR-Based Chromatography Readouts: Indispensable Tools to “Translate” Analytical Features into Molecular Structures. Cells.

[84] Gao D., Cho C.W., Kim J.H., Lee E.J., Kim C.T., Kang J.S. (2020). A New HPLC Method for the Analysis of Puerarin for Quality Control of the Extract of Pueraria Lobate Stem and Puerarin Cream. J. Pharm. Sci..

[85] Gao D., Kim J.H., Kim C.T., Jeong W.S., Kim H.M., Sim J., Kang J.S., Attanzio A. (2021). Molecular Sciences Evaluation of Anti-Melanogenesis Activity of Enriched Pueraria Lobata Stem Extracts and Characterization of Its Phytochemical Components Using HPLC-PDA-ESI-MS/MS. Int. J. Mol. Sci..

[86] Qu L., Song K., Zhang Q., Guo J., Huang J. (2020). Simultaneous Determination of Six Isoflavones from Puerariae Lobatae Radix by CPE-HPLC and Effect of Puerarin on Tyrosinase Activity. Molecules.

[87] Shang X., Huang D., Wang Y., Xiao L., Ming R., Zeng W., Cao S., Lu L., Wu Z., Yan H. (2021). Identification of Nutritional Ingredients and Medicinal Components of Pueraria Lobata and Its Varieties Using Uplc-Ms/Ms-Based Metabolomics. Molecules.

[88] Jin L., Li X., Chen X., Chen X., Liu Y., Xu H., Wang Q., Tang Z. (2023). A Study on Puerarin in Situ Gel Eye Drops: Formulation Optimization and Pharmacokinetics on Rabbits by Microdialysis. Int. J. Pharm..

[89] Yang K., Zhang X., Liu D., Wen S., Wu Y., Li T., Tang T., Wang Y., Zou T., Zhao C. (2023). Water Extracts of Pueraria Thomsonii Radix Ameliorates Alcoholic Liver Disease via PI3K/AKT and NOX4/ROS Pathways. J. Funct. Foods.

[90] Baranyika J.B., Bakire S., Shoucheng P., Meihao S., Hirwa H. (2024). Application of the Selected Macroporous Resin for the Separation and Identification of Flavonoids from Chinese Radix Pueraria Lobata by HPLC-Q-TOF-MS. Microchem. J..

[91] Zheng Y., Ren W., Zhang L., Zhang Y., Liu D., Liu Y. (2020). A Review of the Pharmacological Action of Astragalus Polysaccharide. Front. Pharmacol..

[92] Li Z., Wang Y., Xu Q., Ma J., Li X., Tian Y., Wen Y., Chen T. (2023). Ginseng and Health Outcomes: An Umbrella Review. Front. Pharmacol..

[93] Akaberi M., Baharara H., Amiri M.S., Moghadam A.T., Sahebkar A., Emami S.A. (2023). Ginkgo Biloba: An Updated Review on Pharmacological, Ethnobotanical, and Phytochemical Studies. Pharmacol. Res.-Mod. Chin. Med..

[94] Wang L., Liang Q., Lin A., Chen X., Wu Y., Zhang B., Zhang Y., Min H., Wen Y., Song S. (2020). Puerarin Increases Survival and Protects Against Organ Injury by Suppressing NF-ΚB/JNK Signaling in Experimental Sepsis. Front. Pharmacol..

[95] Zeng X.P., Zeng J.H., Lin X., Ni Y.H., Jiang C.S., Li D.Z., He X.J., Wang R., Wang W. (2021). Puerarin Ameliorates Caerulein-Induced Chronic Pancreatitis via Inhibition of MAPK Signaling Pathway. Front. Pharmacol..

[96] Wang D., Bu T., Li Y., He Y., Yang F., Zou L. (2022). Pharmacological Activity, Pharmacokinetics, and Clinical Research Progress of Puerarin. Antioxidants.

[97] Bai Y.L., Han L.L., Qian J.H., Wang H.Z. (2022). Molecular Mechanism of Puerarin Against Diabetes and Its Complications. Front. Pharmacol..

[98] Shao M., Ye C., Bayliss G., Zhuang S. (2021). New Insights Into the Effects of Individual Chinese Herbal Medicines on Chronic Kidney Disease. Front. Pharmacol..

[99] Tang F., Yan H.L., Wang L.X., Xu J.F., Peng C., Ao H., Tan Y.Z. (2021). Review of Natural Resources with Vasodilation: Traditional Medicinal Plants, Natural Products, and Their Mechanism and Clinical Efficacy. Front. Pharmacol..

[100] Zhou Y.X., Zhang H., Peng C. (2021). Effects of Puerarin on the Prevention and Treatment of Cardiovascular Diseases. Front. Pharmacol..

[101] Li Z., Fan Y., Huang C., Liu Q., Huang M., Chen B., Peng Z., Zhu W., Ding B. (2022). Efficacy and Safety of Puerarin Injection on Acute Heart Failure: A Systematic Review and Meta-Analysis. Front. Cardiovasc. Med..

[102] Xu H., Yu S., Lin C., Dong D., Xiao J., Ye Y., Wang M. (2024). Roles of Flavonoids in Ischemic Heart Disease: Cardioprotective Effects and Mechanisms against Myocardial Ischemia and Reperfusion Injury. Phytomedicine.

[103] Li R., Liang T., He Q., Guo C., Xu L., Zhang K., Duan X. (2013). Puerarin, Isolated from Kudzu Root (Willd.), Attenuates Hepatocellular Cytotoxicity and Regulates the GSK-3β/NF-ΚB Pathway for Exerting the Hepatoprotection against Chronic Alcohol-Induced Liver Injury in Rats. Int. Immunopharmacol..

[104] Liu Y.S., Yuan M.H., Zhang C.Y., Liu H.M., Liu J.R., Wei A.L., Ye Q., Zeng B., Li M.F., Guo Y.P. (2021). Puerariae Lobatae Radix Flavonoids and Puerarin Alleviate Alcoholic Liver Injury in Zebrafish by Regulating Alcohol and Lipid Metabolism. Biomed. Pharmacother..

[105] Yang M., Xia L., Song J., Hu H., Zang N., Yang J., Zou Y., Wang L., Zheng X., He Q. (2023). Puerarin Ameliorates Metabolic Dysfunction-Associated Fatty Liver Disease by Inhibiting Ferroptosis and Inflammation. Lipids Health Dis..

[106] Xiao B., Sun Z., Cao F., Wang L., Liao Y., Liu X., Pan R., Chang Q. (2017). Brain Pharmacokinetics and the Pharmacological Effects on Striatal Neurotransmitter Levels of Pueraria Lobata Isoflavonoids in Rat. Front. Pharmacol..

[107] Gao M., Zhang Z., Lai K., Deng Y., Zhao C., Lu Z., Geng Q. (2022). Puerarin: A Protective Drug against Ischemia-Reperfusion Injury. Front. Pharmacol..

[108] Liang W., Li X., Wang H., Nie K., Meng Q., He J., Zheng C. (2022). Puerarin: A Potential Therapeutic for SARS-CoV-2 and Hantavirus Co-Infection. Front. Immunol..

[109] Lv J., Shi S., Zhang B., Xu X., Zheng H., Li Y., Cui X., Wu H., Song Q. (2022). Role of Puerarin in Pathological Cardiac Remodeling: A Review. Pharmacol. Res..

[110] Jiang Z., Cui X., Qu P., Shang C., Xiang M., Wang J. (2022). Roles and Mechanisms of Puerarin on Cardiovascular Disease: A Review. Biomed. Pharmacother..

[111] Yan J., Honglei Y., Yun W., Sheng D., Yun H., Anhua Z., Na F., Min L., Dandan S., Jing W. (2022). Puerarin Ameliorates Myocardial Remodeling of Spontaneously Hypertensive Rats through Inhibiting TRPC6-CaN-NFATc3 Pathway. Eur. J. Pharmacol..

[112] Qin W., Guo J., Gou W., Wu S., Guo N., Zhao Y., Hou W. (2022). Molecular Mechanisms of Isoflavone Puerarin against Cardiovascular Diseases: What We Know and Where We Go. Chin. Herb. Med..

[113] Peng Y., Wang L., Zhao X., Lai S., He X., Fan Q., He H., He M. (2022). Puerarin Attenuates Lipopolysaccharide-Induced Myocardial Injury via the 14-3-3γ/PKCε Pathway Activating Adaptive Autophagy. Int. Immunopharmacol..

[114] Peng Y., Wang L., Zhang Z., He X., Fan Q., Cheng X., Qiao Y., Huang H., Lai S., Wan Q. (2022). Puerarin Activates Adaptive Autophagy and Protects the Myocardium against Doxorubicin-Induced Cardiotoxicity via the 14–3-3γ/PKCε Pathway. Biomed. Pharmacother..

[115] Xu J., Tian Z., Li Z., Du X., Cui Y., Wang J., Gao M., Hou Y. (2023). Puerarin-Tanshinone IIA Suppresses Atherosclerosis Inflammatory Plaque via Targeting Succinate/HIF-1α/IL-1β Axis. J. Ethnopharmacol..

[116] Hao R., Ge J., Li F., Jiang Y., Sun-Waterhouse D., Li D. (2022). MiR-34a-5p/Sirt1 Axis: A Novel Pathway for Puerarin-Mediated Hepatoprotection against Benzo(a)Pyrene. Free Radic. Biol. Med..

[117] Keskin Alkaç Z., Ahmet Korkak F., Dağoğlu G., Akdeniz İncili C., Dağoğlu Hark B., Tanyıldızı S. (2022). Puerarin Mitigates Oxidative Injuries, Opening of Mitochondrial Permeability Transition Pores and Pathological Damage Associated with Liver and Kidney in Xanthium Strumarium-Intoxicated Rats. Toxicon.

[118] HU Y., WANG S., WU L., YANG K., YANG F., YANG J., HU S., YAO Y., XIA X., LIU Y. (2023). Puerarin Inhibits Inflammation and Lipid Accumulation in Alcoholic Liver Disease through Regulating MMP8. Chin. J. Nat. Med..

[119] Xu B., Wang H., Chen Z. (2021). Puerarin Inhibits Ferroptosis and Inflammation of Lung Injury Caused by Sepsis in LPS Induced Lung Epithelial Cells. Front. Pediatr..

[120] Zhang P., Zhang Y., Wang L., Wang X., Xu S., Zhai Z., Wang C., Cai H. (2022). Reversal of NADPH Oxidase-Dependent Early Oxidative and Inflammatory Responses in Chronic Obstructive Pulmonary Disease by Puerarin. Oxid. Med. Cell Longev..

[121] Peng Z.T., Liu H. (2022). Puerarin Attenuates LPS-Induced Inflammatory Injury in Gastric Epithelial Cells by Repressing NLRP3 Inflammasome-Mediated Apoptosis. Toxicol. In Vitro.

[122] Li X., Wang J., Yan J., He J.C., Li Y., Zhong Y. (2024). Additive Renal Protective Effects between Arctigenin and Puerarin in Diabetic Kidney Disease. Biomed. Pharmacother..

[123] Hou B., Ma P., Yang X., Zhao X., Zhang L., Zhao Y., He P., Du G., Qiang G. (2024). In Silico Prediction and Experimental Validation to Reveal the Protective Mechanism of Puerarin against Excessive Extracellular Matrix Accumulation through Inhibiting Ferroptosis in Diabetic Nephropathy. J. Ethnopharmacol..

[124] Zeng X., Chen B., Wang L., Sun Y., Jin Z., Liu X., Ouyang L., Liao Y. (2023). Chitosan@Puerarin Hydrogel for Accelerated Wound Healing in Diabetic Subjects by MiR-29ab1 Mediated Inflammatory Axis Suppression. Bioact. Mater..

[125] Song X., Wang W., Ding S., Wang Y., Ye L., Chen X., Ma H. (2022). Exploring the Potential Antidepressant Mechanisms of Puerarin: Anti-Inflammatory Response via the Gut-Brain Axis. J. Affect. Disord..

[126] Liu X., Sui X., Zhang Y., Yue R., Yin S. (2023). Efficacy of Puerarin in Rats with Focal Cerebral Ischemia through Modulation of the SIRT1/HIF-1α/VEGF Signaling Pathway and Its Effect on Synaptic Plasticity. Heliyon.

[127] Liu X., Huang R., Wan J. (2023). Puerarin: A Potential Natural Neuroprotective Agent for Neurological Disorders. Biomed. Pharmacother..

[128] Lin S.P., Zhu L., Shi H., Ye S., Li Q., Yin X., Xie Q., Xu Q., Wei J.X., Mei F. (2023). Puerarin Prevents Sepsis-Associated Encephalopathy by Regulating the AKT1 Pathway in Microglia. Phytomedicine.

[129] Zhou S., Li Y., Hong Y., Zhong Z., Zhao M. (2023). Puerarin Protects against Sepsis-Associated Encephalopathy by Inhibiting NLRP3/Caspase-1/GSDMD Pyroptosis Pathway and Reducing Blood-Brain Barrier Damage. Eur. J. Pharmacol..

[130] Liu T., Su K., Cai W., Ao H., Li M. (2023). Therapeutic Potential of Puerarin against Cerebral Diseases: From Bench to Bedside. Eur. J. Pharmacol..

[131] Chen H., Hu X., Lan Y., Chen S., Xiang X., Tan Y., Zeng G., Guo Z., Li K., Zhang J. (2022). Puerarin Promotes Apoptosis and Senescence of Bladder Cancer Cells. J. Funct. Foods.

[132] Ma R., Zhao L., Zhao Y., Li Y. (2022). Puerarin Action on Stem Cell Proliferation, Differentiation and Apoptosis: Therapeutic Implications for Geriatric Diseases. Phytomedicine.

[133] Li T., Shi C., Mi Z., Xu H., Xu J., Wang L., Zhang X. (2022). Biocompatible Puerarin Injectable-Hydrogel Using Self-Assembly Tetrapeptide for Local Treatment of Osteoarthritis in Rats. J. Drug Deliv. Sci. Technol..

[134] Qiu Z., Li L., Huang Y., Shi K., Zhang L., Huang C., Liang J., Zeng Q., Wang J., He X. (2022). Puerarin Specifically Disrupts Osteoclast Activation via Blocking Integrin-Β3 Pyk2/Src/Cbl Signaling Pathway. J. Orthop. Translat.

[135] Li B., Wang Y., Gong S., Yao W., Gao H., Liu M., Wei M. (2022). Puerarin Improves OVX-Induced Osteoporosis by Regulating Phospholipid Metabolism and Biosynthesis of Unsaturated Fatty Acids Based on Serum Metabolomics. Phytomedicine.

[136] Huang W., Guo Y., Han X., Xie X. (2023). Effect and Mechanisms of Puerarin on the Treatment of Postmenopausal Osteoporosis: A Preliminary Pre-Clinical Study. Asian J. Surg..

[137] Meng F., Guo B., Ma Y.-q., Li K.-w., Niu F.-j. (2022). Puerarin: A Review of Its Mechanisms of Action and Clinical Studies in Ophthalmology. Phytomedicine.

[138] Dong Y., Ding Y.Y., Gao W.P. (2024). Puerarin Alleviates Hyperosmotic Stress-Induced Oxidative Stress, Inflammation, Apoptosis and Barrier Damage of Human Corneal Epithelial Cells by Targeting SIRT1/NLRP3 Signaling. Toxicol. In Vitro.

[139] Xu B., Li J., Chen X., Kou M. (2022). Puerarin Attenuates Cisplatin-Induced Apoptosis of Hair Cells through the Mitochondrial Apoptotic Pathway. Biochim. Biophys. Acta Mol. Cell Res..

[140] He L., Wu X., Zhang X., Li X., Lin X., Huang Y., Wu J. (2022). Puerarin Protects against H2O2-Induced Apoptosis of HTR-8/SVneo Cells by Regulating the MiR-20a-5p/VEGFA/Akt Axis. Placenta.

[141] Wu S., Snajdrova R., Moore J.C., Baldenius K., Bornscheuer U.T. (2021). Biocatalysis: Enzymatic Synthesis for Industrial Applications. Angew. Chem. Int. Ed..

[142] Scherer M., Fleishman S.J., Jones P.R., Dandekar T., Bencurova E. (2021). Computational Enzyme Engineering Pipelines for Optimized Production of Renewable Chemicals. Front. Bioeng. Biotechnol..

[143] Nam K., Shao Y., Major D.T., Wolf-Watz M. (2024). Perspectives on Computational Enzyme Modeling: From Mechanisms to Design and Drug Development. ACS Omega.

[144] Dobrzynska M., Napierala M., Florek E. (2020). Flavonoid Nanoparticles: A Promising Approach for Cancer Therapy. Biomolecules.

[145] Liga S., Paul C., Moacă E.A., Péter F. (2024). Niosomes: Composition, Formulation Techniques, and Recent Progress as Delivery Systems in Cancer Therapy. Pharmaceutics.

[146] Ranjbar S., Emamjomeh A., Sharifi F., Zarepour A., Aghaabbasi K., Dehshahri A., Sepahvand A.M., Zarrabi A., Beyzaei H., Zahedi M.M. (2023). Lipid-Based Delivery Systems for Flavonoids and Flavonolignans: Liposomes, Nanoemulsions, and Solid Lipid Nanoparticles. Pharmaceutics.

[147] Chen T., Liu W., Xiong S., Li D., Fang S., Wu Z., Wang Q., Chen X. (2019). Nanoparticles Mediating the Sustained Puerarin Release Facilitate Improved Brain Delivery to Treat Parkinson’s Disease. ACS Appl. Mater. Interfaces.

[148] Yan J., Guan Z.Y., Zhu W.F., Zhong L.Y., Qiu Z.Q., Yue P.F., Wu W.T., Liu J., Huang X. (2020). Preparation of Puerarin Chitosan Oral Nanoparticles by Ionic Gelation Method and Its Related Kinetics. Pharmaceutics.

[149] Han Q., Chen K., Su C., Liu X., Luo X. (2021). Puerarin Loaded PLGA Nanoparticles: Optimization Processes of Preparation and Anti-Alcohol Intoxication Effects in Mice. AAPS PharmSciTech.

[150] Qiang S., Gu L., Kuang Y., Zhao M., You Y., Han Q. (2023). Changes in the Content of Puerarin-PLGA Nanoparticles in Mice under the Influence of Alcohol and Analysis of Their Antialcoholism. J. Appl. Biomater. Funct. Mater..

